# Statistical inferences under step stress partially accelerated life testing based on multiple censoring approaches using simulated and real-life engineering data

**DOI:** 10.1038/s41598-023-39170-x

**Published:** 2023-08-01

**Authors:** Ahmadur Rahman, Mustafa Kamal, Shahnawaz Khan, Mohammad Faisal Khan, Manahil SidAhmed Mustafa, Eslam Hussam, Mintodê Nicodème Atchadé, Aned Al Mutairi

**Affiliations:** 1 Department of Statistics and Operations Research, Aligarh Muslim University, Aligarh, India; 2 Department of Basic Sciences, College of Science and Theoretical Studies, Saudi Electronic University, 32256 Dammam, Saudi Arabia; 3 Faculty of Engineering, Design and Information and Communications Technology, Bahrain Polytechnic, Isa Town, Bahrain; 4 Department of Basic Sciences, College of Science and Theoretical Studies, Saudi Electronic University, 11673 Riyadh, Saudi Arabia; 5 Department of Statistics, Faculty of Science, University of Tabuk, Tabuk, Saudi Arabia; 6 Department of Mathematics, Faculty of Science, Helwan University, Cairo, Egypt; 7 National Higher School of Mathematics Genius and Modelization, National University of Sciences, Technologies, Engineering and Mathematics, Abomey, Republic of Benin; 8 Department of Mathematical Sciences, College of Science, Princess Nourah Bint Abdulrahman University, P.O. Box 84428, 11671 Riyadh, Saudi Arabia

**Keywords:** Software, Statistics

## Abstract

Evaluating the lifespan distribution of highly reliable commodities under regular use is exceedingly difficult, time consuming, and extremely expensive. As a result of its ability to provide more failure data faster and at a lower experimental cost, accelerated life testing has become increasingly important in life testing studies. In this article, we concentrate on parametric inference for step stress partially life testing utilizing multiple censored data based on the Tampered Random Variable model. Under normal stress circumstances, the lifespan of the experimental units is assumed to follow the Nadarajah–Haghighi distribution, with and being the shape and scale parameters, respectively. Maximum likelihood estimates for model parameters and acceleration factor are developed using multiple censored data. We build asymptotic confidence intervals for the unknown parameters using the observed Fisher information matrix. To demonstrate the applicability of the different methodologies, an actual data set based on the timings of subsequent failures of consecutive air conditioning system failures for each member of a Boeing 720 jet aircraft fleet is investigated. Finally, thorough simulation studies utilizing various censoring strategies are performed to evaluate the estimate procedure performance. Several sample sizes were studied in order to investigate the finite sample features of the considered estimators. According to our numerical findings, the values of mean squared errors and average asymptotic confidence intervals lengths drop as sample size increases. Furthermore, when the censoring level is reduced, the considered estimates of the parameters approach their genuine values.

## Introduction

Today’s modern goods are incredibly trustworthy and reliable due to recent scientific advances, innovation, and developments based on computers, automation, and simulations. When a product is very reliable, obtaining failure data through an ordinary life test takes a long time; however, accelerated life testing (ALT) may be used to get a product's dependability life in a short amount of time. This type of test entails submitting test objects to stress settings that shorten its lifetime in comparison to what it would be under normal circumstances. Increased stress speeds up the failure time in ALTs. This means that the amount of time it takes for a product to fail is influenced by the stress. Nelson^1^, Meeker et al.^2^, Kamal^3^, Saxena et al.^4^, El-Din et al.^5^, Rahman et al.^6^ and Han and Bai^7^ provide further information about ALTs.

In general, the acceleration factor in ALT should be well-known, or there should be a well-established model indicating the relationship between life and stress levels. For new things or goods, however, these models do not exist. In such instances, engineers have successfully used partially ALTs to compute the acceleration factor, allowing them to extrapolate accelerated data to normal conditions. Constant-stress and step-stress PALTs (abbreviated as CSPALT and SSPALT) are the two most common categories of PALTs (abbreviated as CSPALT and SSPALT) are depending on how a stress is imposed. In a CSPALT model, each sample of items is subjected to both normal and accelerated levels of constant stress until all units fail or the test is terminated for some reason, such as a censoring method. In SSPALT, a sample of products is first evaluated under regular usage settings for a pre-set amount of time, and then the surviving items are examined under accelerated test conditions until the test is ended for some reason, such as a censoring scheme. Several authors have tackled SSPALT analysis thus far; for example, Goel^8^ proposed the tampered random variable (TRV) model for SSPALT. DeGroot and Goel^9^ investigated SSPALT under Bayesian decision framework based on the TRV model. Bai and Chung^10^, Bai et al.^11^, and Rahman et al.^12^ all examine SSPALT when using alternative life distribution and censoring methods.

The exponential distribution is used as a reference model in statistics, reliability, and life testing assessments due to its lack of memory property. The exponential distribution, on the other hand, is limited to describing only the constant hazard rate. To get around these constraints, Nadarajah and Haghighi^13^ proposed the Nadarajah–Haghighi (NH) distribution, which is an extension of the exponential distribution. In their investigation, they determined that the density function of the NH distribution always has a zero mode. Furthermore, its hazard function can be increasing, decreasing, or constant, and its density function can be monotonically lowering while the hazard rate function is increasing. Because of all of these enticing qualities, the NH distribution may indeed be considered a feasible alternative to the Weibull, Gamma, and exponentiated exponential distributions. In a recent work based on the NH distribution, MirMostafaee et al.^14^ produced the best unbiased linear estimates of the parameters of the NH distribution using moments of upper record values. Selim^15^, Sana and Faizan^16^ offered a brief overview and comparison of frequentist estimating strategies, as well as Bayesian estimates (BE) generated from various loss functions and gamma priors. Kamal et al.^17^ studied a variety of statistical and mathematical features, as well as the maximum likelihood estimation (MLE) technique for parameter estimation, after expanding the NH distribution to a four-parameter distribution. Minic^18^ examined many methods for estimating parameters, based on their biases and mean square errors (MSEs). Kamal et al.^19^ used SSALT to estimate the MLEs of NH distribution parameters.

Number of reasons including time limits, cost savings, and so on. Type-I and type-II censorship are the two most frequently used censoring strategies. In time censoring, also known as type-I censoring, the exam is cancelled after a specified amount of time. Failure censoring, also known as type-II censoring, allows the testing process to be ended after a certain number of failure observations of items. Type-I and type-II censorings do not permit the removal of testing items from a test at any time other than the test completion time. Other censoring techniques, such as progressive censoring and multiple censoring strategies, can be used to address this problem. In progressive censoring, during the test, numerous surviving units are constantly removed at each pre-determined time or failure point until the greatest pre-determined time or failure point is achieved. Multiply censoring is a generalization of traditional and progressive censoring that allows all units in a life test to be removed at any time throughout the test for any reasons, making it more convenient, Wang^20^. This scenario is prevalent in situations when many censoring levels are logically present, as is often the case in many applications in life assessment and survival analysis.

So far, numerous researchers have investigated progressive censored data under SSPALT, but there has been relatively little work on multiply censored data. In SSPALT, Wang et al.^21^ used multiply censored data to produce MLEs of the parameters of the Weibull distribution and the AF. Jia et al.^22^ estimated the reliability using MLEs and Bayes parameter estimates and investigated how to generate confidence intervals for reliability under a multiple censoring scheme. In the presence of multiple censored data, Hassan and Zaky^23^ and Bantan et al.^24^ estimated the Shannon entropy of the inverse Weibull and the inverse Lomax distribution respectively and then used the MLE approach to provide point and confidence interval estimates of parameters. On the basis of multiple censored data and a CSPALT, Alam et al.^25^ and Nassr and Elharoun^26^ developed MLEs of unknown parameters of exponentiated exponential and exponentiated Weibull distributions respectively. For an ALT with k increasing stress levels that is terminated by a progressive censoring strategy, Kamal^27^ produced maximum likelihood estimates of the generalized Pareto distribution parameters. In partially constant-stress accelerated life tests with multiple Type-II censored data, Abushal^28^ used maximum likelihood and Bayes estimation methods to estimate the exponentiated Weibull life time distribution. Using the MLE approach to estimate the parameters of the NH distribution under SSPALT using AT-II PHCS, Kamal et al.^29^ proposed two optimum test procedures based on the A and D optimality. Alam and Ahmed^30^ used AT-II PHCS to explore the MLEs of a Generalized Inverted Exponential distribution under SSPALT. Kamal^31^ explored a hybrid system and employing the MLE approach to estimate parameters of the power linear hazard rate distribution from progressive hybrid censored masked data. For more details see Abd-Elfattah et al.^32^, Nassar et al.^33^, Yousef et al.^34^ and Hassan et al.^35^.

Censoring is the termination of a life experiment before all of the units have failed.

Nonetheless, despite its relevance, the estimation of NH distribution and acceleration factor (AF) parameters under SSPALT for multiple censored data remains an unexplored problem, as far as we know. This work addresses that gap by examining the problem of SSPALT in the context of multiple censored data. Rest of the paper is organized as: In “Modeling SSPALT with MCS” section, we describe the TRV model under basic SSLT and establish the NH baseline lifespan CDF, PDF and RF. In “Inferences under SSPALT with MCS” section, using multiple censored data, in our statistical framework, we compute the MLEs of the parameters $\alpha ,\beta$ and $\theta$, where, $\theta > 1$ denotes the AF. Based on the observed Fisher information matrix, the two-sided approximate confidence intervals (ACIs) of the parameters $\alpha ,\beta$ and $\theta$ are then addressed. To demonstrate the applicability of the various methodologies, an actual data set based on air conditioning system failure times for each member of a Boeing 720 jet aircraft fleet is evaluated in “Real engineering application” section. A thorough numerical analysis is performed, illustrating the positive behavior of the derived estimates over a wide range of sample sizes in “Simulation study” section.

## Modeling SSPALT with MCS

Let $Y$ is a nonnegative random variable distributed according to NH distribution with scale parameter $\beta$ and shape parameter $\alpha$, denoted as NH ($\alpha ,\beta$), then its probability density function (PrDF), cumulative distribution function (CDF) and the survival function (SF) are as follows:

1 $$f\left( {y;\alpha ,\beta } \right) = \alpha \beta \left( {1 + \beta y} \right)^{\alpha - 1} exp\left[ {1 - \left( {1 + \beta y} \right)^{\alpha } } \right],\quad y > 0,\alpha ,\beta > 0$$

2 $$F\left( {y;\alpha ,\beta } \right) = 1 - exp\left[ {1 - \left( {1 + \beta y} \right)^{\alpha } } \right],\quad y > 0,\alpha ,\beta > 0$$

3 $$R\left( y \right) = exp\left[ {1 - \left( {1 + \beta y} \right)^{\alpha } } \right],\quad y > 0,\alpha ,\beta > 0.$$

**Figure 1:**
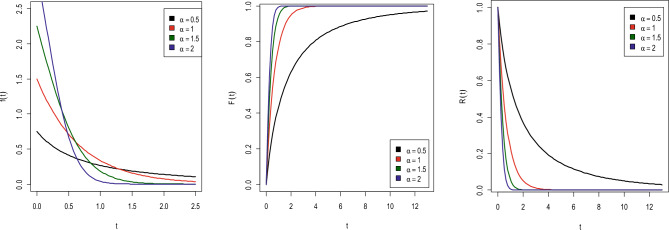
PrDF, CDF, and SF of NH ($\alpha ,\beta$).

Different shapes of PrDF, CDF, and SF that were created using different input values of parameters are displayed in Fig. 1.

As a special instance of the NH distribution, when $\alpha = 1$, an exponential distribution can be produced. It offers closed versions of survival and hazard rate functions, such as that of the Weibull distribution, which makes it an excellent alternative for lifetime data investigators.

In SSPALT, all testing items are first allocated to be tested under ordinary usage settings until a pre-set stress change time $\tau$, following which any remaining survivals that have not failed by time $\tau$ are moved to be tested under accelerated conditions. The effect of stress transition from normal to accelerated condition may be explained by multiplying the remaining lifetime by the inverse of the acceleration factor. The following is a theoretical calculation of the item’s total lifetime $T$ under accelerated conditions:

4 $$T = \left\{ {\begin{array}{*{20}l} Y \hfill & {if\;Y \le \tau } \hfill \\ {\tau + \theta^{ - 1} \left( {Y - \tau } \right)} \hfill & {if\;Y > \tau } \hfill \\ \end{array} } \right.$$

where $Y$ denotes the item’s lifespan under ordinary usage settings and $\theta > 1$ denotes the AF, which is often depending on applied stress. We can now explain the PDF, CDF, and RF of total life $T$ of the objects using the model provided in Eq. (4) as follows:

5 $$f\left( t \right) = \left\{ {\begin{array}{*{20}l} 0 \hfill & {if\;t \le 0} \hfill \\ {f_{1} \left( {t;\alpha ,\beta } \right) = \alpha \beta \left( {1 + \beta t} \right)^{\alpha - 1} exp\left[ {1 - \left( {1 + \beta t} \right)^{\alpha } } \right]} \hfill & {if\;0 < t \le \tau } \hfill \\ {f_{2} \left( t \right) = \alpha \beta \theta \left[ {1 + \beta \left( {\tau + \theta \left( {t - \tau } \right)} \right)} \right]^{\alpha - 1} exp\left[ {1 - \left\{ {1 + \beta \left( {\tau + \theta \left( {t - \tau } \right)} \right)} \right\}^{\alpha } } \right]} \hfill & {if\;t > \tau } \hfill \\ \end{array} } \right.$$

6 $$F(t) = \left\{ {\begin{array}{*{20}l} 0 \hfill & {if\;t \le 0} \hfill \\ {F_{1} \left( {t;\alpha ,\beta } \right) = 1 - exp\left[ {1 - \left( {1 + \beta t} \right)^{\alpha } } \right]} \hfill & {if\;0 < t \le \tau } \hfill \\ {F_{2} \left( t \right) = 1 - exp\left[ {1 - \left\{ {1 + \beta \left( {\tau + \theta \left( {t - \tau } \right)} \right)} \right\}^{\alpha } } \right]} \hfill & {if\;t > \tau } \hfill \\ \end{array} } \right.$$

7 $$R(t) = \left\{ {\begin{array}{*{20}l} 0 \hfill & {if\;t \le 0} \hfill \\ {R_{1} \left( {t;\alpha ,\beta } \right) = exp\left[ {1 - \left( {1 + \beta t} \right)^{\alpha } } \right]} \hfill & {if\;0 < t \le \tau } \hfill \\ {R_{2} \left( t \right) = exp\left[ {1 - \left\{ {1 + \beta \left( {\tau + \theta \left( {t - \tau } \right)} \right)} \right\}^{\alpha } } \right]} \hfill & {if\;t > \tau } \hfill \\ \end{array} } \right.$$

Assume we’re dealing with an SSPALT based on MCS. Assume the test is based on only two stress levels, $X_{1}$ representing regular working conditions and $X_{2}$ representing accelerated conditions, where $X_{2}$ is larger than $X_{1}$. This life testing consists of $n$ objects that are both identical and independent in nature. Under each of the stress levels $X_{1}$ and $X_{2}$, at least one failure should occur. The failure times of the items at each of the stress levels $X_{1}$ and $X_{2}$ are determined by the NH ($\alpha ,\beta$) presented by (1). All items in the sample of size $n$ are now allocated to be tested at stress level $X_{1}$ with a known number $r_{1}$ of failures and a corresponding number $m_{1}$ of multiply censored items till time $\tau$. The test will now be continued by testing all of the items from $n$ that have not failed or been censored up to time $\tau$ at the stress $X_{2}$ with the pre-requisite $r_{2}$ number of failures and the associated number of multiply censored items $m_{2}$ until all of the test items have failed or been censored.

## Inferences under SSPALT with MCS

In this section, the MLE method is utilized to estimate the model parameters and the acceleration factor. This method is more consistent and efficient, providing estimates with greater statistical precision and expressing uncertainty using confidence limits.

Assume that $t_{1,f} ,t_{2,f} , \ldots ,t_{{r_{1} ,f}}$ are the failure timings of $r_{1}$ units that have failed under normal condition $X_{1}$, as well as $m_{1}$ censored units at periods $t_{1,s} ,t_{2,s} , \ldots ,t_{{m_{1} ,s}}$. We further suppose that $t_{1,f} ,t_{2,f} , \ldots ,t_{{r_{2} ,f}}$ are the $r_{2}$ failure times at accelerated condition $X_{2}$ with $m_{2}$ censored units with censoring times $t_{1,s} ,t_{2,s} , \ldots ,t_{{m_{2} ,s}}$. Now, for multiply censored data, the likelihood function under SSALT may be stated as follows Wang et al.^21^:

8 $$L\left( {t,\alpha ,\beta , \theta } \right) = \mathop \prod \limits_{i = 1}^{{r_{1} }} f\left( {t_{i,f} } \right)\mathop \prod \limits_{j = 1}^{m1} \left[ {1 - F\left( {t_{j,s} } \right)} \right]\mathop \prod \limits_{k = 1}^{{r_{2} }} f\left( {t_{k,f} } \right)\mathop \prod \limits_{l = 1}^{{m_{2} }} \left[ {1 - F\left( {t_{l,s} } \right)} \right]$$

The log-likelihood function $l = L\left( {t,\alpha ,\beta , \theta } \right)$ based on MCS under SSPALT corresponding to Eq. (8) after substituting the values of $f\left( {t_{i,f} } \right)$, $f\left( {t_{k,f} } \right)$, $F\left( {t_{j,s} } \right)$ and $F\left( {t_{l,s} } \right)$ can be expressed as:

9 $$\begin{aligned} & l \propto r_{1} {\text{Log}}\left( \alpha \right) + r_{1} {\text{Log}}\left( \beta \right) + r_{2} {\text{Log}}\left( \alpha \right) + r_{2} {\text{Log}}\left( \beta \right) + r_{2} {\text{Log}}\left( \theta \right) \\ & \quad + \;\left( {\alpha - 1} \right)\mathop \sum \limits_{i = 1}^{{r_{1} }} {\text{Log}}\left[ {1 + \beta t_{i} } \right] + \mathop \sum \limits_{i = 1}^{{r_{1} }} {\text{Log}}\left[ {1 - \left( {1 + \beta t_{i} } \right)^{\alpha } } \right] + \left( {\alpha - 1} \right)\mathop \sum \limits_{k = 1}^{{r_{2} }} {\text{Log}}\left[ {1 + \beta A_{k} } \right] \\ & \quad + \;\mathop \sum \limits_{j = 1}^{{m_{1} }} \left( {1 - \left( {1 + \beta t_{j} } \right)^{\alpha } } \right) + \mathop \sum \limits_{k = 1}^{{r_{2} }} \left( {1 - \left( {1 + \beta A_{k} } \right)^{\alpha } } \right) + \mathop \sum \limits_{l = 1}^{{m_{2} }} \left( {1 - \left( {1 + \beta A_{l} } \right)^{\alpha } } \right) \\ \end{aligned}$$

where $\left( {\tau + \theta \left( { - \tau + t_{k} } \right)} \right) = A_{k}$ and $\left( {\tau + \theta \left( { - \tau + t_{l} } \right)} \right) = A_{l}$. Now, the likelihood equations may be derived by calculating partial derivatives of Eq. (9) with respect to $\alpha ,\beta$ and $\theta$ as:

10 $$\begin{aligned} \frac{\partial l}{{\partial \alpha }} & = \frac{{r_{1} }}{\alpha } + \frac{{r_{2} }}{\alpha } + \mathop \sum \limits_{i = 1}^{{r_{1} }} {\text{Log}}\left[ {1 + \beta t_{i} } \right] + \mathop \sum \limits_{k = 1}^{{r_{2} }} {\text{Log}}\left[ {1 + \beta A_{k} } \right] \\ & \quad + \;\mathop \sum \limits_{i = 1}^{{r_{1} }} - \frac{{{\text{Log}}\left[ {1 + \beta t_{i} } \right]\left( {1 + \beta t_{i} } \right)^{\alpha } }}{{1 - \left( {1 + \beta t_{i} } \right)^{\alpha } }} + \mathop \sum \limits_{j = 1}^{{m_{1} }} - {\text{Log}}\left[ {1 + \beta t_{j} } \right]\left( {1 + \beta t_{j} } \right)^{\alpha } \\ & \quad + \;\mathop \sum \limits_{k = 1}^{{r_{2} }} - {\text{Log}}\left[ {1 + \beta A_{k} } \right]\left( {1 + \beta \left( {A_{k} } \right)} \right)^{\alpha } + \mathop \sum \limits_{l = 1}^{{m_{2} }} - {\text{Log}}\left[ {1 + \beta A_{l} } \right]\left( {1 + \beta A_{l} } \right)^{\alpha } \\ \end{aligned}$$

11 $$\begin{aligned} \frac{\partial l}{{\partial \beta }} & = \frac{{r_{1} }}{\beta } + \frac{{r_{2} }}{\beta } + \left( { - 1 + \alpha } \right)\mathop \sum \limits_{i = 1}^{{r_{1} }} \frac{{t_{i} }}{{1 + \beta t_{i} }} + \mathop \sum \limits_{i = 1}^{{r_{1} }} \frac{{\alpha t_{i} \left( {1 + \beta t_{i} } \right)^{ - 1 + \alpha } }}{{ - 1 + \left( {1 + \beta t_{i} } \right)^{\alpha } }} \\ & \quad + \;\mathop \sum \limits_{j = 1}^{{m_{1} }} - \alpha t_{j} \left( {1 + \beta t_{j} } \right)^{ - 1 + \alpha } + \left( { - 1 + \alpha } \right)\mathop \sum \limits_{k = 1}^{{r_{2} }} \frac{{A_{k} }}{{1 + \beta A_{k} }} \\ & \quad + \;\mathop \sum \limits_{k = 1}^{{r_{2} }} \alpha \left( {1 + \beta A_{k} } \right)^{ - 1 + \alpha } \left( {\left( { - 1 + \theta } \right)\tau - \theta t_{k} } \right) + \mathop \sum \limits_{l = 1}^{{m_{2} }} \alpha \left( {1 + \beta A_{l} } \right)^{ - 1 + \alpha } \left( {\left( { - 1 + \theta } \right)\tau - \theta t_{l} } \right) = 0 \\ \end{aligned}$$

12 $$\begin{aligned} \frac{\partial l}{{\partial \theta }} & = \frac{{r_{2} }}{\theta } + \left( { - 1 + \alpha } \right)\mathop \sum \limits_{k = 1}^{{r_{2} }} \frac{{\beta \left( { - \tau + t_{k} } \right)}}{{1 + \beta A_{k} }} \\ & \quad + \;\mathop \sum \limits_{k = 1}^{{r_{2} }} \alpha \beta \left( {1 + \beta A_{k} } \right)^{ - 1 + \alpha } \left( {\tau - t_{k} } \right) + \mathop \sum \limits_{l = 1}^{{m_{2} }} \alpha \beta \left( {1 + \beta A_{l} } \right)^{ - 1 + \alpha } \left( {\tau - t_{l} } \right). \\ \end{aligned}$$

The ML estimates of the parameters, say $\hat{\alpha },\hat{\beta }$ and $\hat{\theta }$, may be derived by solving Eq. (10)–(12) with regard to $\alpha ,\beta$ and $\theta$ respectively. These equations are extremely complex, and they cannot be solved analytically. To solve these simultaneous equations, a numerical iteration approach such as a generic method named as Nelder–Mead Method is advised. In this paper, we utilized the *Optim*() function of R Software.

Because the precise sample distribution of the ML estimates cannot be determined in closed form, the estimated confidence intervals for the parameters $\alpha ,\beta$ and $\theta$ are derived by utilizing the approximate distributions of their ML estimates, which is required to compute the Fisher information matrix. Because the predicted information matrix is excessively complex and necessitates numerical integration, the observed information matrix is produced. The asymptotic distribution of ML estimates of $\alpha ,\beta$ and $\theta$ is given as $\left( {\left( {\hat{\alpha } - \alpha } \right),\left( {\hat{\beta } - \beta } \right), \left( {\hat{\theta } - \theta } \right)} \right) \to N\left( {0,I^{ - 1} \left( {\alpha ,\beta , \theta } \right)} \right)$, where I represent 3 × 3 observed information matrix given in the following equation and the elements of I are given in the below.

13 $$I = \left[ {\begin{array}{*{20}c} { - \frac{{\partial^{2} \ell }}{{\partial \alpha^{2} }}} & { - \frac{{\partial^{2} \ell }}{\partial \alpha \partial \beta }} & { - \frac{{\partial^{2} \ell }}{\partial \alpha \partial \theta }} \\ { - \frac{{\partial^{2} \ell }}{\partial \beta \partial \alpha }} & { - \frac{{\partial^{2} \ell }}{{\partial \beta^{2} }}} & { - \frac{{\partial^{2} \ell }}{\partial \beta \partial \theta }} \\ { - \frac{{\partial^{2} \ell }}{\partial \theta \partial \alpha }} & { - \frac{{\partial^{2} \ell }}{\partial \theta \partial \beta }} & { - \frac{{\partial^{2} \ell }}{{\partial \theta^{2} }}} \\ \end{array} } \right] = \left[ {\begin{array}{*{20}c} {I_{11} } & {I_{12} } & {I_{13} } \\ {I_{21} } & {I_{22} } & {I_{23} } \\ {I_{31} } & {I_{32} } & {I_{33} } \\ \end{array} } \right]$$

And $I^{ - 1} \left( {\alpha ,\beta , \theta } \right)$ represent the variance–covariance matrix and may be approximated by the inverse of the information matrix I, which contains the unknown parameters $\alpha ,\beta$ and $\theta$. Due to the consistency of the ML estimators, the parameters are substituted by the necessary MLEs to obtain an estimated $I^{ - 1} \left( {\alpha ,\beta , \theta } \right)$, which is given by $\hat{I}^{ - 1} \left( {\hat{\alpha },\hat{\beta }, \hat{\theta }} \right)$ as follows:

14 $$\begin{aligned} \hat{I}^{ - 1} \left( {\hat{\alpha },\hat{\beta }, \hat{\theta }} \right) & = \left[ {\begin{array}{*{20}c} { - \frac{{\partial^{2} \ell }}{{\partial \alpha^{2} }}} & { - \frac{{\partial^{2} \ell }}{\partial \alpha \partial \beta }} & { - \frac{{\partial^{2} \ell }}{\partial \alpha \partial \theta }} \\ { - \frac{{\partial^{2} \ell }}{\partial \beta \partial \alpha }} & { - \frac{{\partial^{2} \ell }}{{\partial \beta^{2} }}} & { - \frac{{\partial^{2} \ell }}{\partial \beta \partial \theta }} \\ { - \frac{{\partial^{2} \ell }}{\partial \theta \partial \alpha }} & { - \frac{{\partial^{2} \ell }}{\partial \theta \partial \beta }} & { - \frac{{\partial^{2} \ell }}{{\partial \theta^{2} }}} \\ \end{array} } \right]_{{\left( {\hat{\alpha },\hat{\beta },{ }\hat{\theta }} \right) }}^{ - 1} \\ & = \left[ {\begin{array}{*{20}c} {var.\left( {\hat{\alpha }} \right)} & {cov.\left( {\hat{\alpha }\hat{\beta }} \right)} & {cov.\left( {\hat{\alpha }\hat{\theta }} \right)} \\ {cov.\left( {\hat{\beta }\hat{\alpha }} \right)} & {var.\left( {\hat{\beta }} \right)} & {cov.\left( {\hat{\beta }\hat{\theta }} \right)} \\ {cov.\left( {\hat{\theta }\hat{\alpha }} \right)} & {cov.\left( {\hat{\theta }\hat{\beta }} \right)} & {var.\left( {\hat{\theta }} \right)} \\ \end{array} } \right]. \\ \end{aligned}$$

Now, the estimated $100\left( {1 - \psi } \right)$ percent double-sided confidence bounds with $Z_{\psi /2}$ as an upper $\left( {\psi /2} \right){\text{th}}$ percentile of standard normal variate for $\alpha ,\beta$ and $\theta$ are presented as follows:

15 $$\begin{aligned} & \hat{\alpha } - Z_{\psi /2} \sqrt {I_{11}^{ - 1} \left( {\hat{\alpha },\hat{\beta }, \hat{\theta }} \right)} \;{\text{and}}\;\hat{\alpha } + Z_{\psi /2} \sqrt {I_{11}^{ - 1} \left( {\hat{\alpha },\hat{\beta }, \hat{\theta }} \right)} \\ & \hat{\beta } - Z_{\psi /2} \sqrt {I_{22}^{ - 1} \left( {\hat{\alpha },\hat{\beta }, \hat{\theta }} \right)} \;{\text{and}}\;\hat{\beta } + Z_{\psi /2} \sqrt {I_{22}^{ - 1} \left( {\hat{\alpha },\hat{\beta }, \hat{\theta }} \right)} \\ & \hat{\theta } - Z_{\psi /2} \sqrt {I_{22}^{ - 1} \left( {\hat{\alpha },\hat{\beta }, \hat{\theta }} \right)} \;{\text{and}}\;\hat{\theta } + Z_{\psi /2} \sqrt {I_{22}^{ - 1} \left( {\hat{\alpha },\hat{\beta }, \hat{\theta }} \right)} . \\ \end{aligned}$$

### Second derivatives

$$\begin{aligned} \frac{{\partial^{2} \ell }}{{\partial \alpha^{2} }} & = - \frac{{r_{1} }}{{\alpha^{2} }} - \frac{{r_{2} }}{{\alpha^{2} }} + \mathop \sum \limits_{i = 1}^{{r_{1} }} - \frac{{{\text{Log}}\left[ {1 + \beta t_{i} } \right]^{2} \left( {1 + \beta t_{i} } \right)^{\alpha } }}{{\left( { - 1 + \left( {1 + \beta t_{i} } \right)^{\alpha } } \right)^{2} }} + \mathop \sum \limits_{j = 1}^{{m_{1} }} - {\text{Log}}\left[ {1 + \beta t_{j} } \right]^{2} \left( {1 + \beta t_{j} } \right)^{\alpha } \\ & \quad + \;\mathop \sum \limits_{k = 1}^{{r_{2} }} - {\text{Log}}\left[ {1 + \beta A_{k} } \right]^{2} \left( {1 + \beta A_{k} } \right)^{\alpha } + \mathop \sum \limits_{l = 1}^{{m_{2} }} - {\text{Log}}\left[ {1 + \beta A_{l} } \right]^{2} \left( {1 + \beta A_{l} } \right)^{\alpha } \\ \end{aligned}$$

$$\begin{aligned} \frac{{\partial^{2} \ell }}{{\partial \beta^{2} }} & = - \frac{{r_{1} }}{{\beta^{2} }} - \frac{{r_{2} }}{{\beta^{2} }} + \left( { - 1 + \alpha } \right)\mathop \sum \limits_{i = 1}^{{r_{1} }} - \frac{{t_{i}^{2} }}{{\left( {1 + \beta t_{i} } \right)^{2} }} + \mathop \sum \limits_{i = 1}^{{r_{1} }} - \alpha t_{i} \left[ {\left( { - 1 + \alpha } \right)t_{i} \left( {1 + \beta t_{i} } \right)^{ - 2 + \alpha } } \right] \\ & \quad + \;\mathop \sum \limits_{j = 1}^{{m_{1} }} - \alpha t_{j} \left[ {\left( { - 1 + \alpha } \right)t_{j} \left( {1 + \beta t_{j} } \right)^{ - 2 + \alpha } } \right] + \left( { - 1 + \alpha } \right)\mathop \sum \limits_{k = 1}^{{r_{2} }} - \frac{{\left( {\tau + \theta \left[ { - \tau + t_{k} } \right]} \right)^{2} }}{{\left( {1 + \beta A_{k} } \right)^{2} }} \\ & \quad + \;\mathop \sum \limits_{k = 1}^{{r_{2} }} - \left( { - 1 + \alpha } \right)\alpha \left( {1 + \beta A_{k} } \right)^{ - 2 + \alpha } \theta \left[ { - \tau + t_{k} } \right]\left( {\tau + \theta \left[ { - \tau + t_{k} } \right]} \right) - \alpha \tau \left[ {\left( { - 1 + \alpha } \right)\left( {1 + \beta A_{k} } \right)^{ - 2 + \alpha } \left( {\tau + \theta \left[ { - \tau + t_{k} } \right]} \right)} \right] \\ & \quad + \;\mathop \sum \limits_{l = 1}^{{m_{2} }} - \left( { - 1 + \alpha } \right)\alpha \left( {1 + \beta A_{l} } \right)^{ - 2 + \alpha } \theta \left[ { - \tau + t_{l} } \right]\left( {\tau + \theta \left[ { - \tau + t_{l} } \right]} \right) - \alpha \tau \left[ {\left( { - 1 + \alpha } \right)\left( {1 + \beta A_{l} } \right)^{ - 2 + \alpha } \left( {\tau + \theta \left[ { - \tau + t_{l} } \right]} \right)} \right] \\ \end{aligned}$$

$$\begin{aligned} \frac{{\partial^{2} \ell }}{{\partial \theta^{2} }} & = - \frac{{r_{2} }}{{\theta^{2} }} + \left( { - 1 + \alpha } \right)\mathop \sum \limits_{k = 1}^{{r_{2} }} \frac{{\beta \left( {\tau - t_{k} } \right)\beta \left[ { - \tau + t_{k} } \right]}}{{\left( {1 + \beta A_{k} } \right)^{2} }} \\ & \quad + \;\mathop \sum \limits_{k = 1}^{{r_{2} }} \alpha \beta \left( {\tau \left[ { - \left( { - 1 + \alpha } \right)\beta \left( {1 + \beta A_{k} } \right)^{ - 2 + \alpha } \left( {\tau - t_{k} } \right)\left] { - t_{k} } \right[ - \left( { - 1 + \alpha } \right)\beta \left( {1 + \beta A_{k} } \right)^{ - 2 + \alpha } \left( {\tau - t_{k} } \right)} \right]} \right) \\ & \quad + \;\mathop \sum \limits_{l = 1}^{{m_{2} }} \alpha \beta \left( {\tau \left[ { - \left( { - 1 + \alpha } \right)\beta \left( {1 + \beta A_{l} } \right)^{ - 2 + \alpha } \left( {\tau - t_{l} } \right)\left] { - t_{l} } \right[ - \left( { - 1 + \alpha } \right)\beta \left( {1 + \beta A_{l} } \right)^{ - 2 + \alpha } \left( {\tau - t_{l} } \right)} \right]} \right) \\ \end{aligned}$$

$$\begin{aligned} \frac{{\partial^{2} \ell }}{\partial \alpha \partial \beta } & = \mathop \sum \limits_{i = 1}^{{r_{1} }} \frac{{t_{i} }}{{1 + \beta t_{i} }} + \mathop \sum \limits_{i = 1}^{{r_{1} }} \frac{{t_{i} \left( {1 + \beta t_{i} } \right)^{ - 1 + \alpha } \left( { - 1 - \alpha {\text{Log}}\left[ {1 + \beta t_{i} } \right] + \left( {1 + \beta t_{i} } \right)^{\alpha } } \right)}}{{\left( { - 1 + \left( {1 + \beta t_{i} } \right)^{\alpha } } \right)^{2} }} \\ & \quad + \;\mathop \sum \limits_{j = 1}^{{m_{1} }} - \left( {1 + \alpha {\text{Log}}\left[ {1 + \beta t_{j} } \right]} \right)t_{j} \left( {1 + \beta t_{j} } \right)^{ - 1 + \alpha } + \mathop \sum \limits_{k = 1}^{{r_{2} }} \frac{{A_{k} }}{{1 + \beta A_{k} }} \\ & \quad + \;\mathop \sum \limits_{k = 1}^{{r_{2} }} \left( {1 + \beta A_{k} } \right)^{ - 1 + \alpha } \left( { - A_{k} + \alpha {\text{Log}}\left[ {1 + \beta A_{k} } \right]\left( {\left( { - 1 + \theta } \right)\tau - \theta t_{k} } \right)} \right) \\ & \quad + \;\mathop \sum \limits_{l = 1}^{{m_{2} }} \left( {1 + \beta A_{l} } \right)^{ - 1 + \alpha } \left( { - A_{l} + \alpha {\text{Log}}\left[ {1 + \beta A_{l} } \right]\left( {\left( { - 1 + \theta } \right)\tau - \theta t_{l} } \right)} \right) \\ \end{aligned}$$

$$\begin{aligned} \frac{{\partial^{2} \ell }}{\partial \alpha \partial \theta } & = \mathop \sum \limits_{k = 1}^{{r_{2} }} \frac{{\beta \left( { - \tau + t_{k} } \right)}}{{1 + \beta A_{k} }} + \mathop \sum \limits_{k = 1}^{{r_{2} }} \left( {1 + \beta A_{k} } \right)^{ - 1 + \alpha } \left( {\alpha \beta {\text{Log}}\left[ {1 + \beta A_{k} } \right]\left( {\tau - t_{k} } \right) - \beta \left[ { - \tau + t_{k} } \right]} \right) \\ & \quad + \mathop \sum \limits_{l = 1}^{{m_{2} }} \left( {1 + \beta A_{l} } \right)^{ - 1 + \alpha } \left( {\alpha \beta {\text{Log}}\left[ {1 + \beta A_{l} } \right]\left( {\tau - t_{l} } \right) - \beta \left[ { - \tau + t_{l} } \right]} \right) \\ \end{aligned}$$

$$\begin{aligned} \frac{{\partial^{2} \ell }}{\partial \theta \partial \beta } & = \left( { - 1 + \alpha } \right)\mathop \sum \limits_{k = 1}^{{r_{2} }} \left( {\frac{{ - \tau + t_{k} }}{{1 + \beta A_{k} }} + \frac{{\beta \left( { - \tau + t_{k} } \right)\left( {\theta \tau - \tau - \theta t_{k} } \right)}}{{\left( {1 + \beta A_{k} } \right)^{2} }}} \right) \\ & \quad + \;\mathop \sum \limits_{k = 1}^{{r_{2} }} \alpha \left( {1 + \beta A_{k} } \right)^{ - 1 + \alpha } \left( {\tau - t_{k} } \right) + \alpha \beta \left( {\tau \left[ {\left( {1 + \beta A_{k} } \right)^{ - 2 + \alpha } \left( {\left( { - 1 + \alpha + \theta } \right)\tau + \left( { - 1 + \alpha } \right)\theta t_{k} } \right) - \alpha \theta \tau \left( {1 + \beta \left[ {\tau + \theta \left[ { - \tau + t_{k} } \right]} \right]} \right)^{ - 2 + \alpha } } \right]} \right. \\ & \quad \left. { - \;t_{k} \left[ { - \left( { - 1 + \alpha } \right)\left( {1 + \beta A_{k} } \right)^{ - 2 + \alpha } \left( {\left( { - 1 + \theta } \right)\tau - \theta t_{k} } \right)} \right]} \right) + \mathop \sum \limits_{l = 1}^{{m_{2} }} \alpha \left( {1 + \beta A_{l} } \right)^{ - 1 + \alpha } \left( {\tau - t_{l} } \right) \\ & \quad + \;\alpha \beta \left( {\tau \left[ { - \left( { - 1 + \alpha } \right)\left( {1 + \beta A_{l} } \right)^{ - 2 + \alpha } \left( {\left( { - 1 + \theta } \right)\tau - \theta t_{l} } \right)} \right] - t_{l} \left[ { - \left( { - 1 + \alpha } \right)\left( {1 + \beta A_{l} } \right)^{ - 2 + \alpha } \left( {\left( { - 1 + \theta } \right)\tau - \theta t_{l} } \right)} \right]} \right) \\ \end{aligned}$$

## Real engineering application

In this section, we employ real data supplied by Proschon^36^ to demonstrate the real engineering application of the estimation approaches offered in this paper. The data set provides the times of successive failures of sequential air conditioning system failures for each member of a Boeing 720 jet aircraft fleet. This data is also saved in R’s npsurv package under the name acfail Wang^37^. The recorded data is given as follows:

1 1 2 3 3 3 3 4 5 5 5 5 5 7 7 7 9 9 10 11 11 11 11 12 12 12 12 13 14 14 14 14 14 14 14 14 15 15 15 16 16 16 18 18 18 18 18 18 20 20 21 21 22 22 22 23 23 23 24 24 25 26 26 27 27 29 29 29 29 30 31 31 32 33 33 34 34 34 35 35 36 36 37 39 39 41 42 43 44 44 44 46 46 47 47 48 49 50 50 51 52 54 54 55 56 56 57 57 57 58 59 59 59 60 61 61 62 62 62 63 65 66 67 67 68 70 70 71 71 72 74 76 77 79 79 80 82 84 85 87 88 90 90 91 95 97 97 98 100 100 101 102 102 104 104 104 106 111 118 118 120 120 130 130 130 134 139 141 142 152 153 156 163 169 176 181 182 184 186 188 191 194 197 201 206 208 208 209 210 216 220 225 230 230 239 246 246 254 261 270 283 310 320 326 359 386 413 438 447 487 493 502 603.

To assess the data’s goodness-of-fit to the NH distribution, we first calculated the NH distribution’s parameters and then the K–S test was utilized. The K-S statistic and their *p* value are then calculated and reported in Table 1 as follows:

**Table 1:** The K–S statistic, *p* value and estimates of the NH distribution’s parameters.

Model	$\hat{\alpha }$	$\hat{\beta }$	K–S Statistics	*p* value
NH distribution	0.7296426	0.0185777	0.04613	0.7552

**Figure 2:**
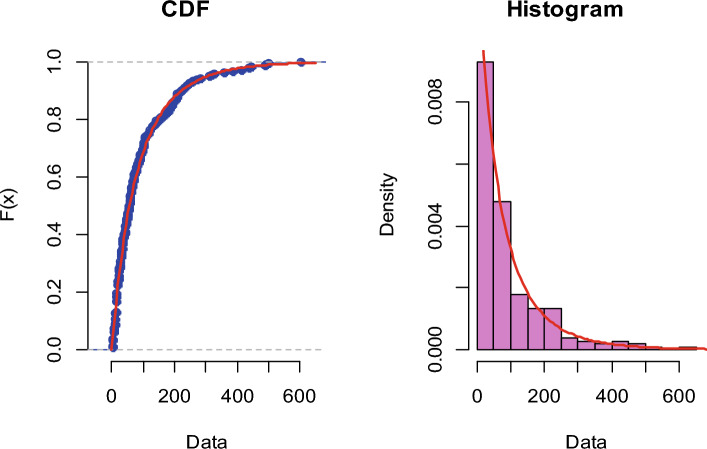
Theoretical CDF of the NH distribution vs the empirical CDF and histogram.

**Figure 3:**
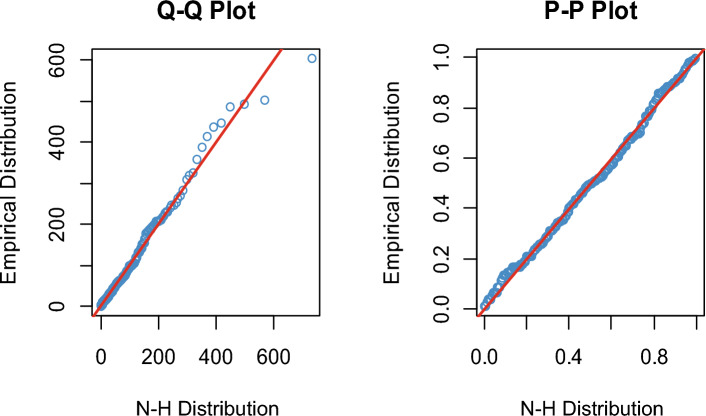
The Q–Q and P–P plots of the supplied actual data set.

The K-S distance is determined to be 0.04613, with an associated *p* value of 0.7552. Since the *p* value is more than 0.05, we cannot reject the null hypothesis, which states that both the theoretical and sample distributions are same. Furthermore, multiple plots are analyzed for the goodness-of-fit test to check further if the data fits the NH distribution. We also illustrate the fitting of the distribution by plotting the estimated cdf, Q–Q and P–P plots of the NH distribution for the supplied real data set. Figure 2 compares the theoretical CDF of the NH distribution to the empirical CDF and histogram. The Q–Q and P–P plots of the supplied actual data set are shown in Fig. 3. The figures and KS test results suggest that the real data set under consideration fits the NH distribution pretty well.

Now, under SSPALT, we assume that the test runs under normal operating condition until the high stress is applied, and then the stress is raised to make the test operate at accelerated condition. We assume that the stress change time $\tau$ is 57 and various censoring levels (CL) are 20%, 30%, and 40%. To reflect multiple censoring strategy, we now delete 20%, 30%, and 40% of data at both stress levels, and the observed data under standard and accelerated stress with the assumed censoring levels are provided in Table 2.

**Table 2:** Multiply censored data at both stress level with $\tau = 57$ and CLs of 20%, 30%, and 40%.

Stress	CL (%)	Data	
Normal	20	Observed	1 1 2 3 3 3 3 4 5 5 5 5 5 7 7 7 9 9 10 11 11 11 11 12 12 12 12 13 14 14 14 14 14 14 14 14 15 15 15 16 16 16 18 18 18 18 18 18 20 20 21 21 22 22 22 23 23 23 24 24 25 26 26 27 27 29 29 29 29 30 31 31 32 33 33 34 34 34 35 35 36 36 37 39 39
		Censored	41 42 43 44 44 44 46 46 47 47 48 49 50 50 51 52 54 54 55 56 56
Accelerated	20	Observed	58 59 59 59 60 61 61 62 62 62 63 65 66 67 67 68 70 70 71 71 72 74 76 77 79 79 80 82 84 85 87 88 90 90 91 95 97 97 98 100 100 101 102 102 104 104 104 106 111 118 118 120 120 130 130 130 134 139 141 142 152 153 156 163 169 176 181 182 184 186 188 191 194 197 201 206 208 208 209 210 216 220
		Censored	225 230 230 239 246 246 254 261 270 283 310 320 326 359 386 413 438 447 487 493 502 603
Normal	30	Observed	1 1 2 3 3 3 3 4 5 5 5 5 5 7 7 7 9 9 10 11 11 11 11 12 12 12 12 13 14 14 14 14 14 14 14 14 15 15 15 16 16 16 18 18 18 18 18 18 20 20 21 21 22 22 22 23 23 23 24 24 25 26 26 27 27 29 29 29 29 30 31 31 32 33 33
		Censored	34 34 34 35 35 36 36 37 39 39 41 42 43 44 44 44 46 46 47 47 48 49 50 50 51 52 54 54 55 56 56
Accelerated	30	Observed	58 59 59 59 60 61 61 62 62 62 63 65 66 67 67 68 70 70 71 71 72 74 76 77 79 79 80 82 84 85 87 88 90 90 91 95 97 97 98 100 100 101 102 102 104 104 104 106 111 118 118 120 120 130 130 130 134 139 141 142 152 153 156 163 169 176 181 182 184 186 188 191
		Censored	194 197 201 206 208 208 209 210 216 220 225 230 230 239 246 246 254 261 270 283 310 320 326 359 386 413 438 447 487 493 502 603
Normal	40	Observed	1 1 2 3 3 3 3 4 5 5 5 5 5 7 7 7 9 9 10 11 11 11 11 12 12 12 12 13 14 14 14 14 14 14 14 14 15 15 15 16 16 16 18 18 18 18 18 18 20 20 21 21 22 22 22 23 23 23 24 24 25 26 26 27 27
		Censored	29 29 29 29 30 31 31 32 33 33 34 34 34 35 35 36 36 37 39 39 41 42 43 44 44 44 46 46 47 47 48 49 50 50 51 52 54 54 55 56 56
Accelerated	40	Observed	58 59 59 59 60 61 61 62 62 62 63 65 66 67 67 68 70 70 71 71 72 74 76 77 79 79 80 82 84 85 87 88 90 90 91 95 97 97 98 100 100 101 102 102 104 104 104 106 111 118 118 120 120 130 130 130 134 139 141 142 152
		Censored	153 156 163 169 176 181 182 184 186 188 191 194 197 201 206 208 208 209 210 216 220 225 230 230 239 246 246 254 261 270 283 310 320 326 359 386 413 438 447 487 493 502 603

We now estimated the MLEs and loglikelihood function values with stress change time $\tau = 57$ and various censoring levels using the real data set reported in Table 2. The findings are computed using the R software, and Table 3 summarizes the resulting MLEs and loglikelihood function values. As per the results in Table 3, the estimates perform better at a 20 percent censoring level than at 30 percent and 40 percent filtering levels. This is apparent since larger data sets yield better results with more precision.

**Table 3:** The MLEs and loglikelihood function values with $\tau = 57$ and CLs of 20%, 30%, and 40%.

Model	CL (%)	− Loglikelihood	$\hat{\alpha }$	$\hat{\beta }$	$\hat{\theta }$
NH distribution	20	**944.8238**	**0.3006398**	**0.0547679**	**2.7774441**
	30	839.7492	0.22741321	0.07540592	4.16437398
	40	726.1117	0.1701904	0.1048783	6.3930727

Significant values are in [bold].

We have now raised the stress change duration from 57 to 71 while keeping the same censoring levels of 20%, 30%, and 40% to test the model's flexibility. Table 4 shows the observed data under standard and accelerated stress, as well as the censoring levels after eliminating 20%, 30%, and 40% of the data for both stress levels.

**Table 4:** Multiply censored data at both stress level with $\tau = 71$ and CLs of 20%, 30%, and 40%.

Stress	CL (%)	Data	
Normal	20	Observed	1 1 2 3 3 3 3 4 5 5 5 5 5 7 7 7 9 9 10 11 11 11 11 12 12 12 12 13 14 14 14 14 14 14 14 14 15 15 15 16 16 16 18 18 18 18 18 18 20 20 21 21 22 22 22 23 23 23 24 24 25 26 26 27 27 29 29 29 29 30 31 31 32 33 33 34 34 34 35 35 36 36 37 39 39 41 42 43 44 44 44 46 46 47 47 48 49 50 50 51 52 54 54
		Censored	55 56 56 57 57 57 58 59 59 59 60 61 61 62 62 62 63 65 66 67 67 68 70 70
Accelerated	20	Observed	72 74 76 77 79 79 80 82 84 85 87 88 90 90 91 95 97 97 98 100 100 101 102 102 104 104 104 106 111 118 118 120 120 130 130 130 134 139 141 142 152 153 156 163 169 176 181 182 184 186 188 191 194 197 201 206 208 208 209 210 216 220 225 230 230 239
		Censored	254 261 270 283 310 320 326 359 386 413 438 447 487 493 502 603
Normal	30	Observed	1 1 2 3 3 3 3 4 5 5 5 5 5 7 7 7 9 9 10 11 11 11 11 12 12 12 12 13 14 14 14 14 14 14 14 14 15 15 15 16 16 16 18 18 18 18 18 18 20 20 21 21 22 22 22 23 23 23 24 24 25 26 26 27 27 29 29 29 29 30 31 31 32 33 33 34 34 34 35 35 36 36 37 39 39 41 42 43 44 44 44
		Censored	46 46 47 47 48 49 50 50 51 52 54 54 55 56 56 57 57 57 58 59 59 59 60 61 61 62 62 62 63 65 66 67 67 68 70 70
Accelerated	30	Observed	72 74 76 77 79 79 80 82 84 85 87 88 90 90 91 95 97 97 98 100 100 101 102 102 104 104 104 106 111 118 118 120 120 130 130 130 134 139 141 142 152 153 156 163 169 176 181 182 184 186 188 191 194 197 201 206 208 208
		Censored	209 210 216 220 225 230 230 239 246 246 254 261 270 283 310 320 326 359 386 413 438 447 487 493 502 603
Normal	40	Observed	1 1 2 3 3 3 3 4 5 5 5 5 5 7 7 7 9 9 10 11 11 11 11 12 12 12 12 13 14 14 14 14 14 14 14 14 15 15 15 16 16 16 18 18 18 18 18 18 20 20 21 21 22 22 22 23 23 23 24 24 25 26 26 27 27 29 29 29 29 30 31 31 32 33 33 34 34 34
		Censored	35 35 36 36 37 39 39 41 42 43 44 44 44 46 46 47 47 48 49 50 50 51 52 54 54 55 56 56 57 57 57 58 59 59 59 60 61 61 62 62 62 63 65 66 67 67 68 70 70
Accelerated	40	Observed	72 74 76 77 79 79 80 82 84 85 87 88 90 90 91 95 97 97 98 100 100 101 102 102 104 104 104 106 111 118 118 120 120 130 130 130 134 139 141 142 152 153 156 163 169 176 181 182 184 186
		Censored	188 191 194 197 201 206 208 208 209 210 216 220 225 230 230 239 246 246 254 261 270 283 310 320 326 359 386 413 438 447 487 493 502 603

Subsequently, we estimated the MLEs and loglikelihood function values with stress change time $\tau = 71$ and various censoring levels using the real data set reported in Table 4. The findings are again computed using the R software, Team^38^ and Table 5 summarizes the resulting MLEs and loglikelihood function values. As per the results in Table 5, we again observed that the estimates perform better at a 20% censoring level than at 30%, and 40% filtering levels.

**Table 5:** The MLEs and loglikelihood function values with $\tau = 71$ and CLs of 20%, 30%, and 40%.

Model	CL (%)	− Loglikelihood	$\hat{\alpha }$	$\hat{\beta }$	$\hat{\theta }$
NH distribution	20	**955.8221**	**0.37524261**	**0.04355366**	**1.77960490**
	30	856.5320	0.27304217	0.06320305	2.58774850
	40	745.2779	0.20317581	0.08857279	3.91300152

Significant values are in [bold].

## Simulation study

This section provides a simulation study to investigate the performance of MLEs of parameters for NH distribution under SSPALT based on multiply censored data. The mean squared error (MSE) is used to compare the performance of point estimates, whereas the CPs are used to compare the performance of ACIs. The data were derived from Eq. (2) using the inverse CDF approach. The quantile function that has been utilized for this task is defined as $t = \left( {\left( {1 - \log (1 - u)} \right)^{1/\alpha } - 1} \right)/\beta$, where *u* is generated from uniform distribution, i.e., $u \sim U\left( {0,1} \right)$. We selected five different sample sizes $n$ = 80, 90, 100, 110, and 120 in order to analyse the nature of the estimates as the sample size increased. We also considered the NH distribution in order to produce data with starting values of the shape parameter $\alpha = 0.2$ and the scale parameter $\beta = 1.6$. The value of acceleration factor is set to $\theta = 2.5$, with two distinct values of stress change time $\tau = 5, 8$ and three different censoring levels (CL) of 20%, 30%, and 40%. The simulation study is developed based on 8,000 multiply censored samples under SSPALT to observe changes in parameter values. The complete steps of the algorithm are detailed below:

i. Using the pre-specified parameter values, generate $n$ random samples from the NH distribution. To do so, first generate *u* from a uniform distribution with the command $u = runif\left( {n, 0, 1} \right)$, and then use the quantile function $t = \left( {\left( {1 - \log (1 - u} \right))^{1/\alpha } - 1} \right)/\beta$ to generate the values of $t = \left( {t_{1} ,t_{2} , \ldots ,t_{n} } \right)$ from an NH distribution.
ii. Now select the number of failures before the stress change time $\tau$ and denote it as $n_{1}$. The generated sample up to time $\tau$ is $t_{1s} = \left( {t_{1} ,t_{2} , \ldots ,t_{{n_{1} }} } \right)$*.* Also select the number of failed items at accelerated condition and denote it as $n_{2}$, where $n_{1} + n_{2} = n$ and the generated sample is given as $t_{2s} = \left( {t_{{n_{1} + 1}} ,t_{{n_{1} + 2}} \ldots ,t_{{n_{1} + n_{2} }} } \right)$
iii. Choose the CL at 20%, 30% and 40% respectively at both normal and accelerated condition. At 20% CL, we have 80% failed items and 20% censored items. So, we have number of failures at normal and accelerated stress level $r_{1} = n_{1} \left( {1 - CL} \right)$ and $r_{2} = n_{2} \left( {1 - CL} \right)$ respectively.
iv. Let $$\delta_{1s,i} = \left\{ {\begin{array}{*{20}l} {1,} \hfill & {i = 1,2, \ldots ,r_{1} } \hfill \\ {0,} \hfill & {i = r_{1} + 1,r_{1} + 2, \ldots ,n_{1} } \hfill \\ \end{array} } \right.$$ and $$\delta_{2s,j} = \left\{ {\begin{array}{*{20}l} {1,} \hfill & {j = 1,2, \ldots ,r_{2} } \hfill \\ {0,} \hfill & {i = r_{2} + 1,r_{2} + 2, \ldots ,n_{2} } \hfill \\ \end{array} } \right.$$
v. Now set $y_{1,i} = \delta_{1s,i} \times t_{1s}$ and $y_{2,j} = \delta_{2s,j} \times \left( {\frac{{\left( {t_{2s} - \tau } \right)}}{\alpha } + \tau } \right)$, thus the generated multiply censored data set is given as: $Y = \left\{ {y_{1,i} ,y_{2,j} ,i = 1,2,...,n_{1} ,j = 1,2,...,n_{2} } \right\}$
vi. Estimate the parameters using the $Optim\left( \right)$ function and the data obtained in steps (i–v).
vii. Repeat the process N times.
viii. Now calculate the mean MLEs, mean MSEs, 95% ACIs and its CPs by using the following formulae.
  $$\begin{aligned} \hat{\alpha } & = \frac{1}{N}\mathop \sum \limits_{i = 1}^{N} \alpha_{i} ; \hat{\beta } = \frac{1}{N}\mathop \sum \limits_{i = 1}^{N} \beta_{i} ; \hat{\theta } = \frac{1}{N}\mathop \sum \limits_{i = 1}^{N} \theta_{i} ; CP\left( {\hat{\alpha }, \hat{\beta }, \hat{\theta }} \right) \\ & = No.\;of\;ACI\;includes\;\left( {\hat{\alpha }, \hat{\beta }, \hat{\theta }} \right)/N \\ \end{aligned}$$
  $$\begin{aligned} & MSE\left( {\hat{\alpha }} \right) = \frac{1}{N}\mathop \sum \limits_{i = 1}^{N} (\hat{\alpha }_{i} - \alpha )^{2} ;\;MSE\left( {\hat{\beta }} \right) = \frac{1}{N}\mathop \sum \limits_{i = 1}^{N} (\hat{\beta }_{i} - \beta )^{2} ; \\ & MSE\left( {\hat{\theta }} \right) = \frac{1}{N}\mathop \sum \limits_{i = 1}^{N} (\hat{\theta }_{i} - \theta )^{2} \\ \end{aligned}$$

**Figure 4:**
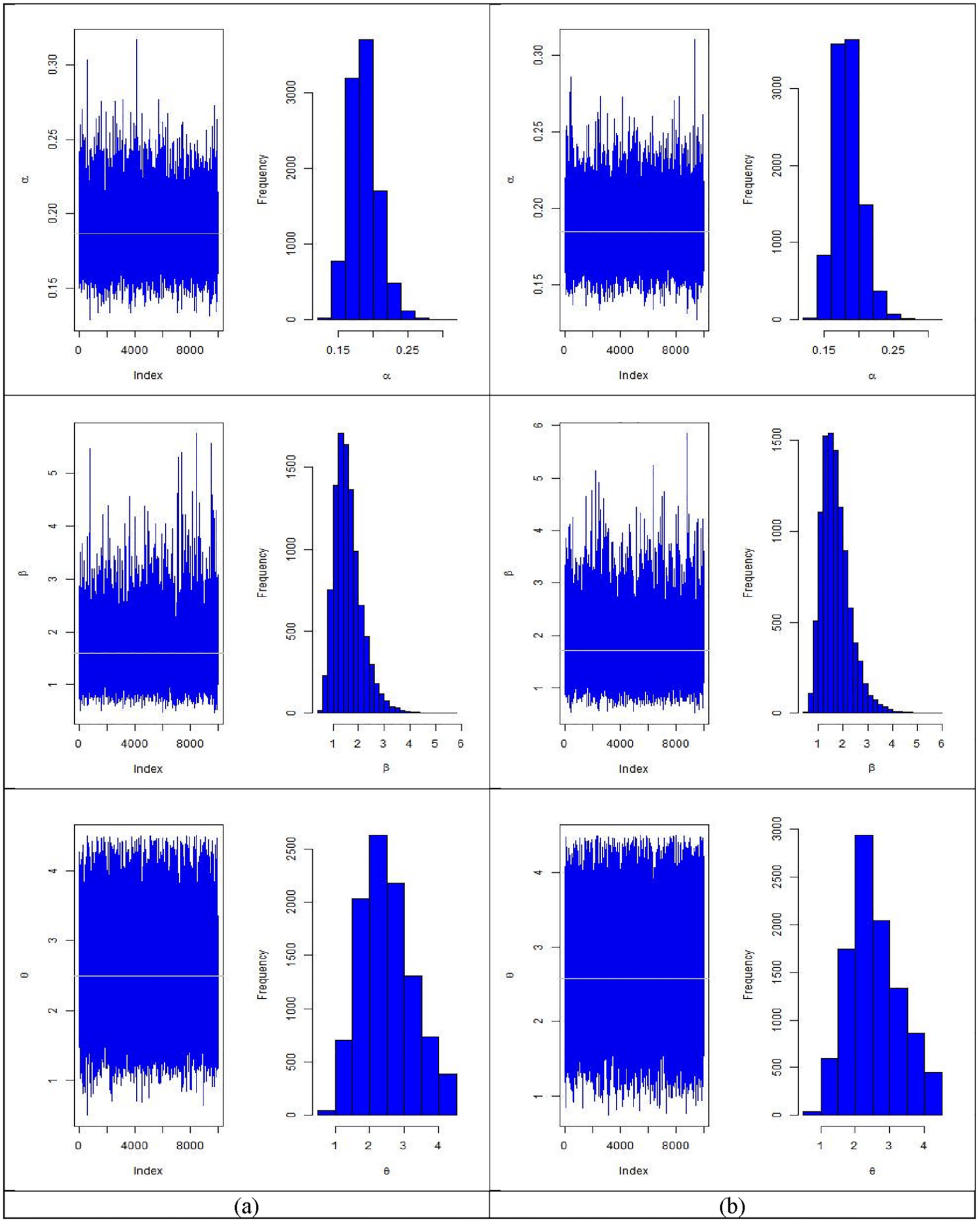
The plots for simulated samples and the histogram of the parameters for $\left( {\alpha ,\beta , \theta } \right)$ with $\left( {\alpha = 0.2,\beta = 1.6,\theta = 2.5,CL = 0.20} \right)$ for (**a**) $\tau = 5$ and (**b**) $\tau = 8$.

**Figure 5:**
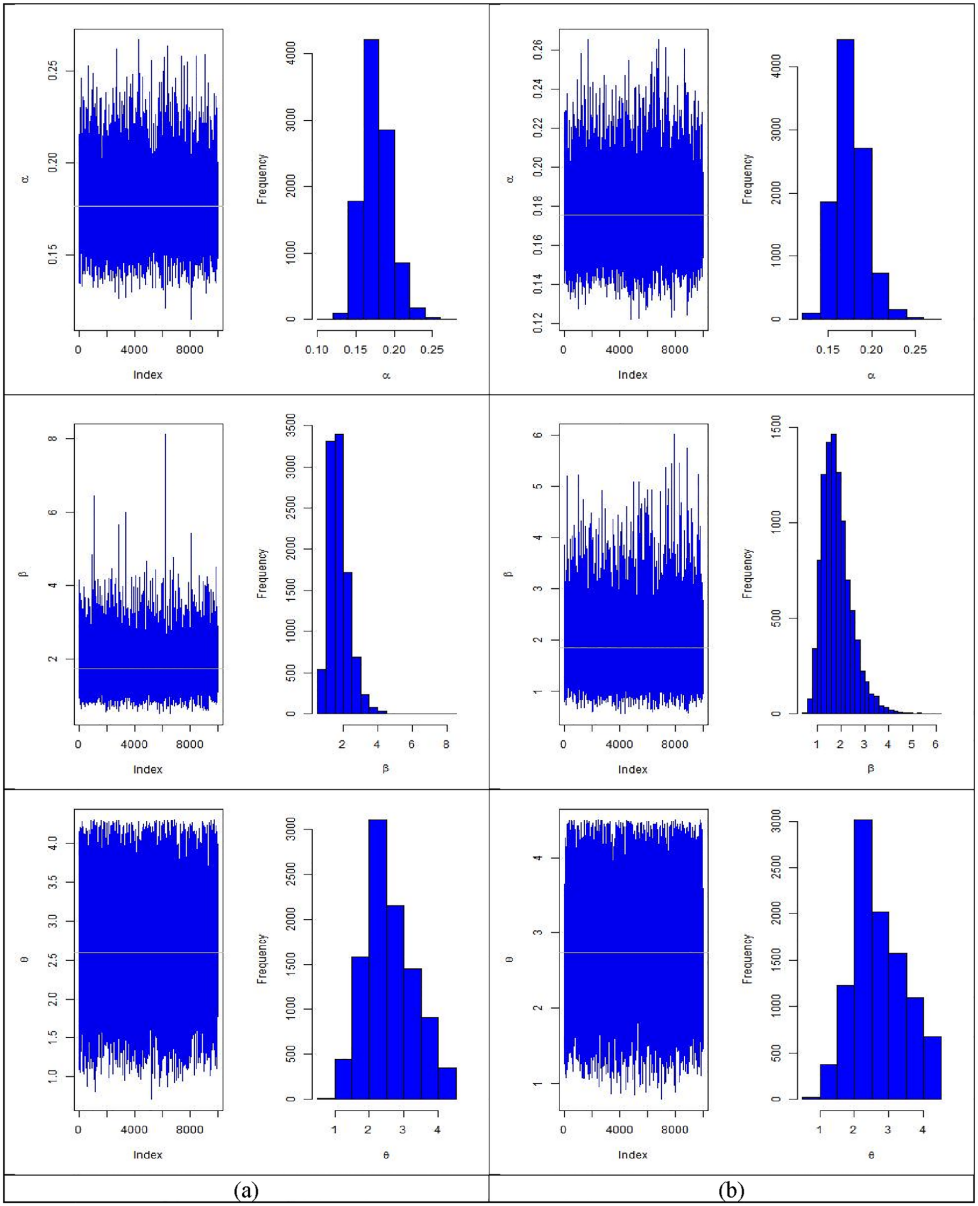
The plots for simulated samples and the histogram of the parameters for $\left( {\alpha ,\beta , \theta } \right)$ with $\left( {\alpha = 0.2,\beta = 1.6,\theta = 2.5,CL = 0.30} \right)$ for (**a**) $\tau = 5$ and (**b**) $\tau = 8$.

**Figure 6:**
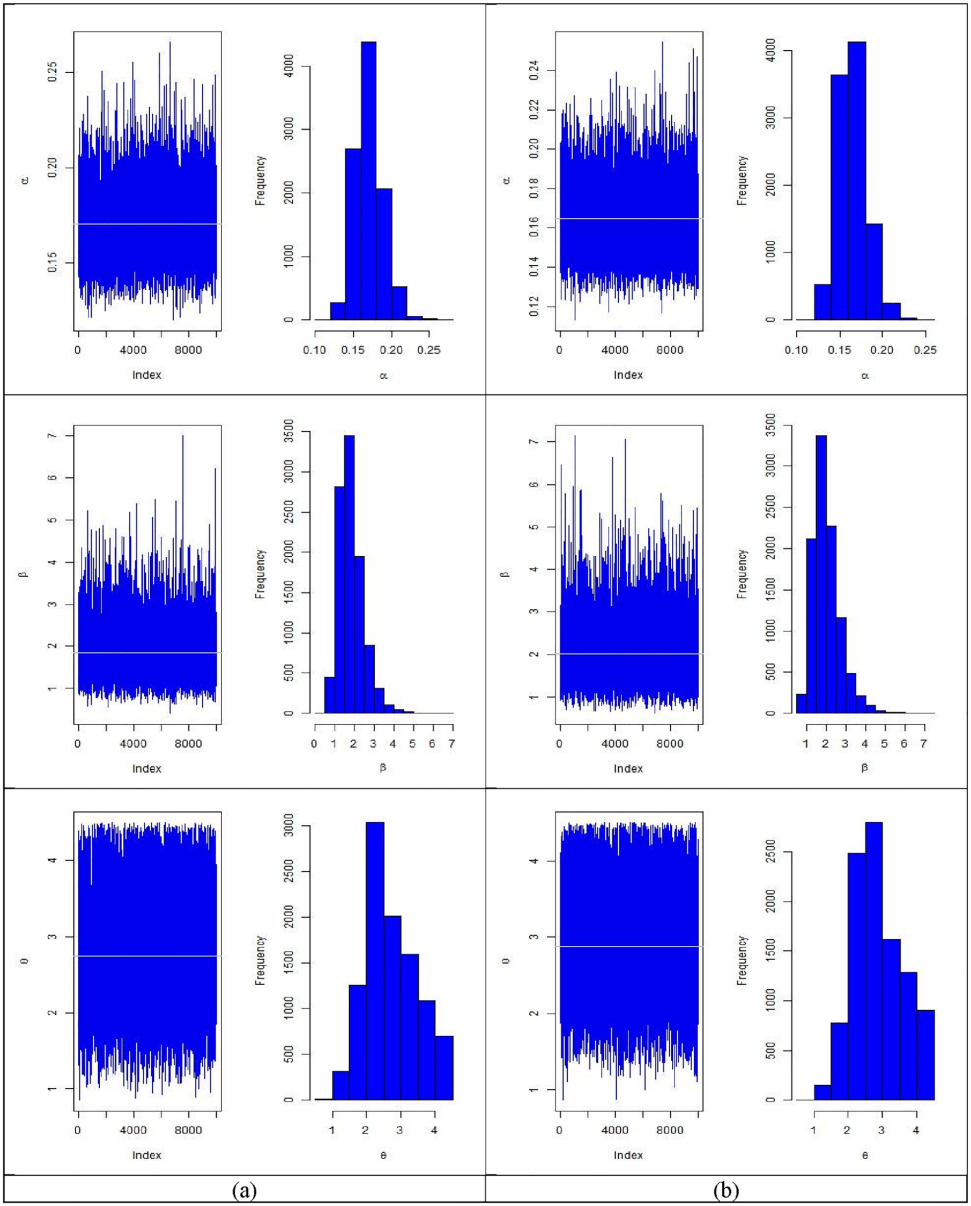
The plots for simulated samples and the histogram of the parameters for $\left( {\alpha ,\beta , \theta } \right)$ with $\left( {\alpha = 0.2,\beta = 1.6,\theta = 2.5,CL = 0.40} \right)$ for (**a**) $\tau = 5$ and (**b**) $\tau = 8$.

In Tables 6, 7, 8, 9, 10 and 11 summarizes MLEs, MSEs, Lower 95% ACI Limit (L95%CL), Upper 95% ACI Limit (U95%CL), 95% ACI length (95%ACIL) and 95% ACI Coverage Probability (95%ACICP) under multiply censored data based on MLE method are presented with $\left( {\alpha = 0.2,\beta = 1.6,\theta = 2.5} \right)$, $\tau = \left( {5, 8} \right)$ and different CL of 20%, 30%, and 40% based on N = 10,000 simulations respectively. Figures 3, 4, 5 and 6 provides the plots for simulated samples and the histogram of the parameters based on N = 10,000 simulations respectively based on different initial values of parameters and distinct values of stress change time.

**Table 6:** MLEs, MSEs, lower 95% ACI limit (L95%CL), upper 95% ACI limit (U95%CL), 95% ACI length (95%ACIL) and 95% ACI coverage probability (95%ACICP) under multiply censored data with $\left( {\alpha = 0.2,\beta = 1.6,\theta = 2.5,CL = 0.20,\tau = 5} \right)$.

*n*	Parameters	MLEs	MSEs	L95%CL	U95%CL	95%ACIL	95%ACICP
80	$\alpha$	0.1674595	0.0205570	0.1271677	0.2077513	0.080583	95.33905
	$\beta$	1.769901	0.737674	0.32406	3.215742	2.891653	95.19905
	$\theta$	2.769546	0.7580471	1.283774	4.255319	2.971515	94.73905
90	$\alpha$	0.1658654	0.0193200	0.1279981	0.2037327	0.075734	95.17905
	$\beta$	1.688259	0.6639709	0.3868759	2.989642	2.60274	95.68904
	$\theta$	2.810845	0.7488733	1.343053	4.278637	2.935555	94.94905
100	$\alpha$	0.1641433	0.0181911	0.1284886	0.199798	0.071309	95.12905
	$\beta$	1.695375	0.6315235	0.4575886	2.933161	2.475548	95.41905
	$\theta$	2.870894	0.7401584	1.420184	4.321605	2.901392	95.42905
110	$\alpha$	0.1658051	0.0175181	0.1314695	0.2001406	0.06867	94.97905
	$\beta$	1.687168	0.6067909	0.4978576	2.876478	2.378597	95.71904
	$\theta$	2.871401	0.7416124	1.41784	4.324961	2.907092	95.65904
120	$\alpha$	0.164657	0.0167029	0.1319192	0.1973949	0.065475	95.24905
	$\beta$	1.7164	0.5776405	0.5842243	2.848575	2.264328	95.64904
	$\theta$	2.876607	0.7224927	1.460521	4.292693	2.832144	95.55904

**Table 7:** MLEs, MSEs, lower 95% ACI Limit (L95%CL), upper 95% ACI limit (U95%CL) and 95% ACI coverage probability (95%ACICP) under multiply censored data with $\left( {\alpha = 0.2,\beta = 1.6,\theta = 2.5,CL = 0.30,\tau = 5} \right)$.

*n*	Parameters	MLEs	MSEs	L95%CL	U95%CL	95%ACIL	95%ACICP
80	$\alpha$	0.171446	0.0213531	0.1295938	0.2132982	0.083704	95.18905
	$\beta$	1.962471	0.820904	0.3534995	3.571443	3.217911	95.50904
	$\theta$	2.725251	0.7837417	1.189118	4.261385	3.072236	95.00905
90	$\alpha$	0.1766426	0.0208124	0.1358502	0.217435	0.081584	95.45905
	$\beta$	1.841277	0.7137327	0.4423609	3.240193	2.797804	95.57904
	$\theta$	2.682347	0.7729612	1.167344	4.197351	3.029977	94.69905
100	$\alpha$	0.1772288	0.0203246	0.1373925	0.2170652	0.079672	95.40905
	$\beta$	1.837299	0.6865213	0.4917177	3.182881	2.691136	95.35905
	$\theta$	2.694221	0.7757881	1.173676	4.214765	3.041059	95.10905
110	$\alpha$	0.1750145	0.0189565	0.1378596	0.2121695	0.074309	95.47905
	$\beta$	1.85638	0.6671368	0.5487923	3.163969	2.615151	95.58904
	$\theta$	2.742036	0.7588724	1.254647	4.229426	2.974749	95.16905
120	$\alpha$	0.175405	0.0181038	0.1399213	0.2108886	0.070967	95.42905
	$\beta$	1.845531	0.6238778	0.6227309	3.068332	2.445577	95.51904
	$\theta$	2.735305	0.7447443	1.275606	4.195004	2.919369	95.11905

**Table 8:** MLEs, MSEs, lower 95% ACI limit (L95%CL), upper 95% ACI limit (U95%CL) and 95% ACI coverage probability (95%ACICP) under multiply censored data with $\left( {\alpha = 0.2,\beta = 1.6,\theta = 2.5,CL = 0.40,\tau = 5} \right)$.

*n*	Parameters	MLEs	MSEs	L95%CL	U95%CL	95%ACIL	95%ACICP
80	$\alpha$	0.1842316	0.0234016	0.1383644	0.2300987	0.091733	95.26905
	$\beta$	2.018154	0.8621193	0.3284004	3.707908	3.379474	95.46905
	$\theta$	2.548723	0.803562	0.9737411	4.123704	3.149931	94.88905
90	$\alpha$	0.1884445	0.0230097	0.1433454	0.2335436	0.090197	95.41905
	$\beta$	2.028738	0.8237137	0.4142595	3.643217	3.228925	95.39905
	$\theta$	2.507637	0.7935419	0.952295	4.062979	3.110653	95.21905
100	$\alpha$	0.1878581	0.0220437	0.1446523	0.2310639	0.086411	95.44905
	$\beta$	2.044117	0.789722	0.4962623	3.591973	3.09568	95.52904
	$\theta$	2.510402	0.7776811	0.9861468	4.034657	3.04848	95.00905
110	$\alpha$	0.1878068	0.0210943	0.1464619	0.2291517	0.082689	95.42905
	$\beta$	2.003293	0.7248494	0.5825885	3.423998	2.841381	95.39905
	$\theta$	2.497154	0.7693558	0.9892164	4.005091	3.015844	95.04905
120	$\alpha$	0.1844116	0.0193433	0.1464987	0.2223245	0.075825	95.24905
	$\beta$	2.011573	0.6981329	0.6432326	3.379914	2.736654	95.54904
	$\theta$	2.569823	0.7482025	1.103346	4.0363	2.932925	94.98905

**Table 9:** MLEs, MSEs, lower 95% ACI limit (L95%CL), upper 95% ACI limit (U95%CL) and 95% ACI coverage probability (95%ACICP) under multiply censored data with $\left( {\alpha = 0.2,\beta = 1.6,\theta = 2.5,CL = 0.20,\tau = 8} \right)$.

*n*	Parameters	MLEs	MSEs	L95%CL	U95%CL	95%ACIL	95%ACICP
80	$\alpha$	0.1695727	0.0213753	0.127677	0.2114684	0.083791	95.25905
	$\beta$	1.642838	0.681189	0.307708	2.977969	2.670234	95.56904
	$\theta$	2.709395	0.7676959	1.204711	4.214079	3.009338	94.47906
90	$\alpha$	0.1707	0.0204468	0.1306242	0.2107757	0.080151	95.31905
	$\beta$	1.62826	0.641043	0.3718155	2.884704	2.512863	95.61904
	$\theta$	2.727482	0.7656334	1.22684	4.228123	3.001253	95.09905
100	$\alpha$	0.167875	0.0189223	0.1307873	0.2049628	0.074175	95.31905
	$\beta$	1.618495	0.5924418	0.4573089	2.779681	2.322349	95.56904
	$\theta$	2.746631	0.7499382	1.276752	4.21651	2.939729	94.83905
110	$\alpha$	0.1694406	0.0185174	0.1331465	0.2057348	0.072588	95.23905
	$\beta$	1.607871	0.5645694	0.5013148	2.714427	2.21309	95.41905
	$\theta$	2.747332	0.7473003	1.282623	4.21204	2.929388	94.78905
120	$\alpha$	0.1703368	0.0177683	0.1355109	0.2051626	0.069651	95.30905
	$\beta$	1.5948	0.5427752	0.5309605	2.658639	2.127657	95.56904
	$\theta$	2.74297	0.7431093	1.286476	4.199464	2.912959	95.08905

**Table 10:** MLEs, MSEs, lower 95% ACI limit (L95%CL), upper 95% ACI limit (U95%CL) and 95% ACI coverage probability (95%ACICP) under multiply censored data with $\left( {\alpha = 0.2,\beta = 1.6,\theta = 2.5,CL = 0.30,\tau = 8} \right)$.

*n*	Parameters	MLEs	MSEs	L95%CL	U95%CL	95%ACIL	95%ACICP
80	$\alpha$	0.177812	0.022881	0.132965	0.22266	0.089694	95.30905
	$\beta$	1.75734	0.751602	0.284201	3.230479	2.946249	95.80904
	$\theta$	2.56948	0.738227	1.122556	4.016405	2.89382	95.01905
90	$\alpha$	0.178401	0.021554	0.136155	0.220648	0.084492	95.34905
	$\beta$	1.73631	0.676127	0.411102	3.061518	2.650389	95.48905
	$\theta$	2.584085	0.738027	1.137552	4.030617	2.893036	95.24905
100	$\alpha$	0.1792	0.020719	0.138591	0.219809	0.081217	95.40905
	$\beta$	1.718056	0.641242	0.461222	2.97489	2.513643	95.81904
	$\theta$	2.565261	0.72391	1.146397	3.984125	2.8377	95.08905
110	$\alpha$	0.175728	0.019147	0.1382	0.213255	0.075054	95.40905
	$\beta$	1.764415	0.611778	0.565329	2.963501	2.398148	95.56904
	$\theta$	2.619841	0.712766	1.222819	4.016862	2.794015	95.03905
120	$\alpha$	0.176476	0.01852	0.140177	0.212776	0.072598	95.04905
	$\beta$	1.745166	0.589055	0.590618	2.899715	2.309074	95.45905
	$\theta$	2.601612	0.699721	1.230159	3.973065	2.742879	95.01905

**Table 11:** MLEs, MSEs, lower 95% ACI limit (L95%CL), upper 95% ACI limit (U95%CL) and 95% ACI coverage probability (95%ACICP) under multiply censored data with $\left( {\alpha = 0.2,\beta = 1.6,\theta = 2.5,CL = 0.40,\tau = 8} \right)$.

*n*	Parameters	MLEs	MSEs	L95%CL	U95%CL	95%ACIL	95%ACICP
80	$\alpha$	0.1872706	0.0251297	0.1380162	0.236525	0.098508	95.48905
	$\beta$	1.836654	0.6234703	0.6146522	3.058656	2.443979	95.62904
	$\theta$	2.469478	0.8019501	0.8976558	4.0413	3.143613	95.20905
90	$\alpha$	0.1870743	0.0231267	0.1417459	0.2324027	0.090656	95.16905
	$\beta$	1.847026	0.7307637	0.4147291	3.279323	2.864565	95.50904
	$\theta$	2.472528	0.7871014	0.9298092	4.015247	3.085407	95.32905
100	$\alpha$	0.1866643	0.0222978	0.1429606	0.230368	0.087407	95.59904
	$\beta$	1.905395	0.7147208	0.5045425	3.306248	2.801677	95.67904
	$\theta$	2.488021	0.7761908	0.9666865	4.009355	3.042638	95.03905
110	$\alpha$	0.1866207	0.0212001	0.1450684	0.228173	0.083104	95.19905
	$\beta$	1.861991	0.6653099	0.557984	3.165999	2.607989	95.39905
	$\theta$	2.4773	0.7553382	0.9968368	3.957763	2.960897	94.72905
120	$\alpha$	0.1866518	0.0203459	0.1467736	0.2265299	0.079756	95.32905
	$\beta$	1.873683	0.7921207	0.3211266	3.42624	3.105082	95.68904
	$\theta$	2.497773	0.7465648	1.034506	3.96104	2.926505	95.13905

**Figure 7:**
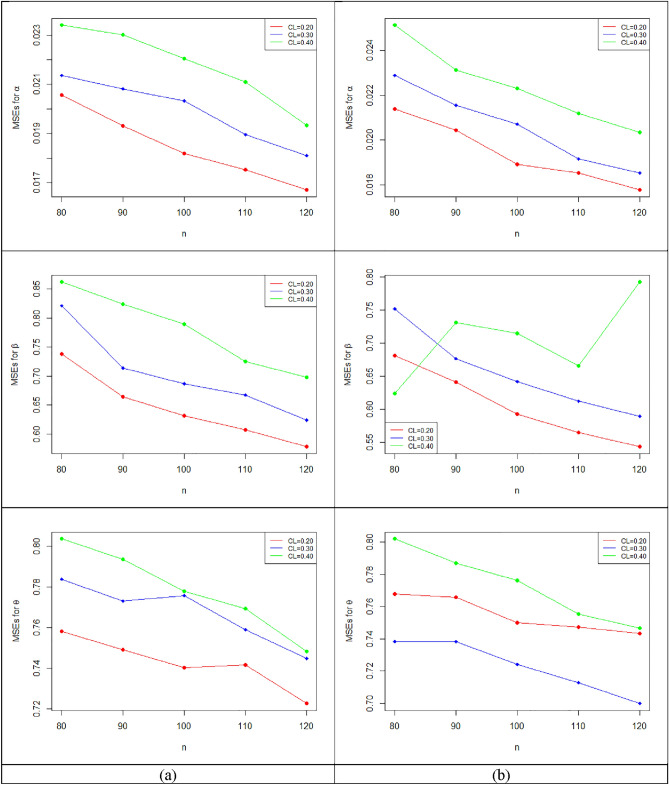
The plots of MSEs of the estimates for $\left( {\alpha ,\beta , \theta } \right)$ with $\left( {\alpha = 0.2,\beta = 1.6,\theta = 2.5} \right)$ for (**a**) $\tau = 5$ and (**b**) $\tau = 8$.

**Figure 8:**
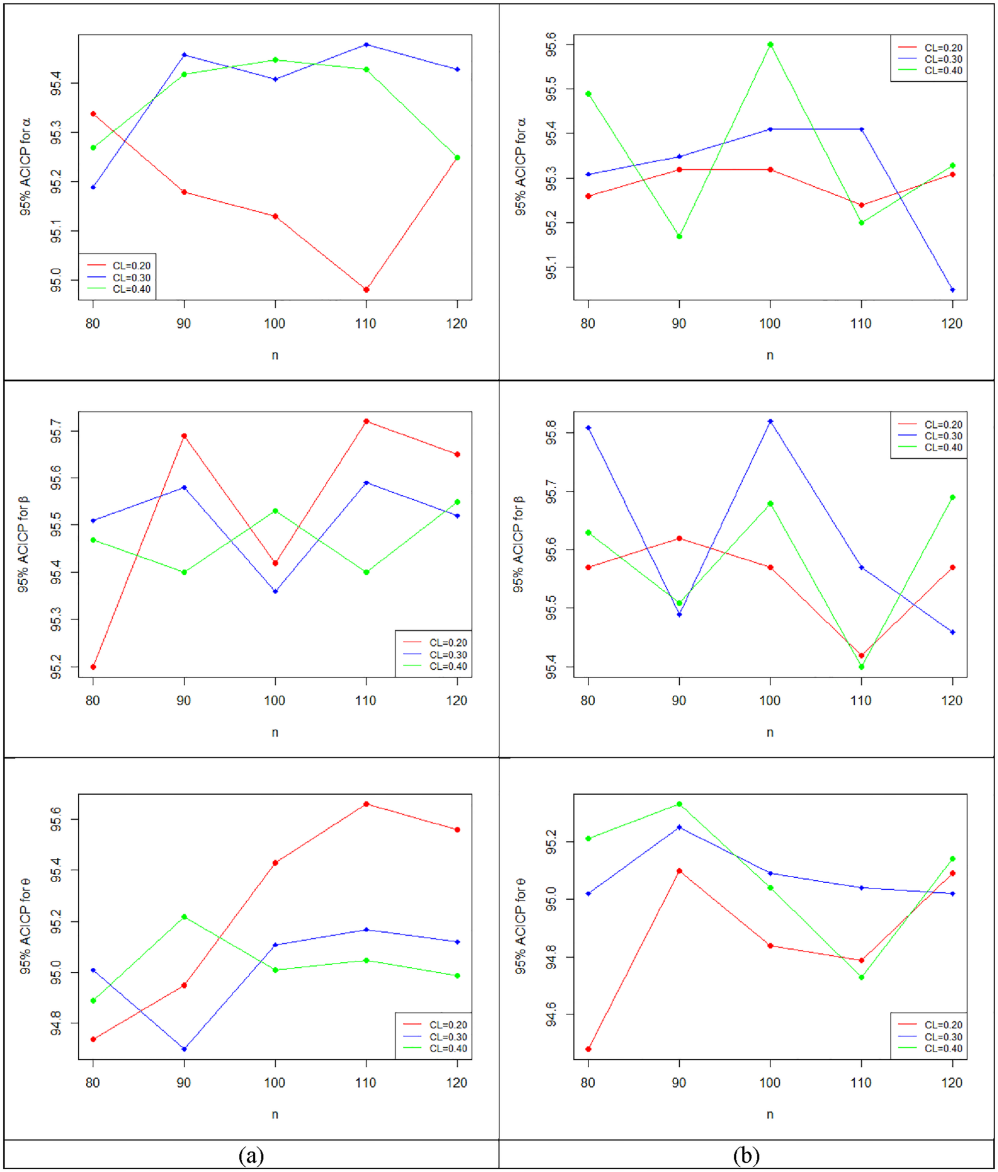
The plots of 95%ACICP of the estimate for $\left( {\alpha ,\beta , \theta } \right)$ with $\left( {\alpha = 0.2,\beta = 1.6,\theta = 2.5} \right)$ for (**a**) $\tau = 5$ and (**b**) $\tau = 8$.

**Figure 9:**
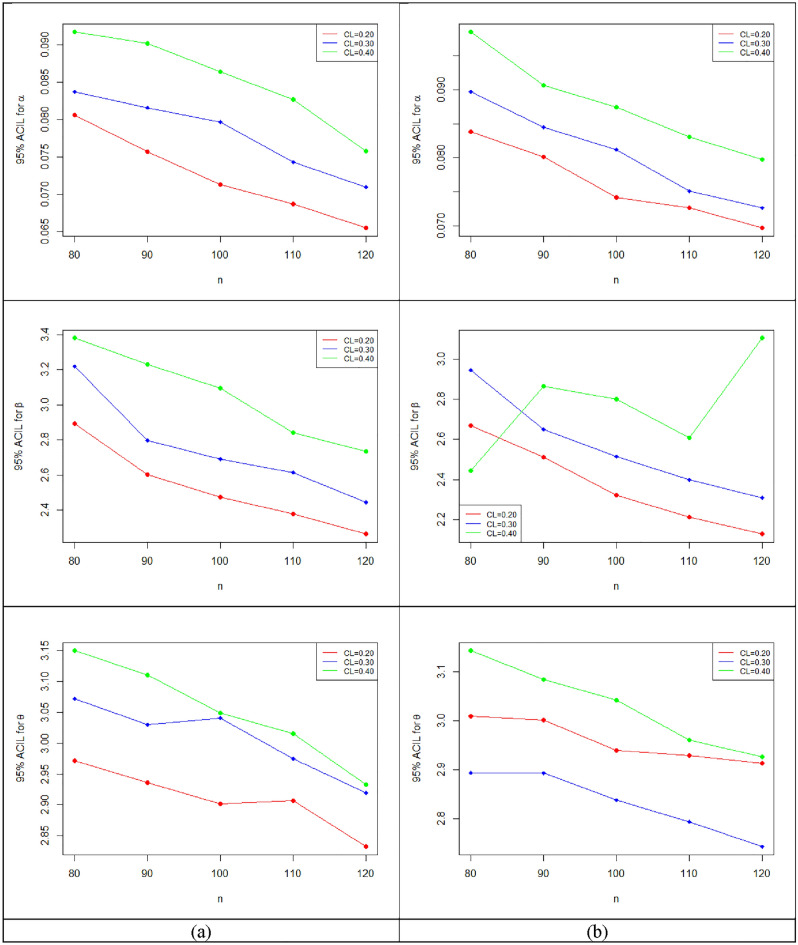
The plots of 95%ACIL of the estimate for $\left( {\alpha ,\beta , \theta } \right)$ with $\left( {\alpha = 0.2,\beta = 1.6,\theta = 2.5} \right)$ for (**a**) $\tau = 5$ and (**b**) $\tau = 8$.

Based on the results in Tables 6, 7, 8, 9, 10 and 11 and Figs. 7, 8 and 9, we can observe that:

i. The MLEs of the parameters $\alpha , \beta$ and $\theta$ based on multiply censored data are moving closer to their true values with decreasing MSEs in all cases as *n* grows.
ii. The values of MSEs and the average length of 95%ACIs fall as *n* grows for fixed values of $\alpha , \beta , \theta$ and $\tau$ while the related 95%ACI coverage probabilities approach 95%.
iii. For fixed values of $\alpha , \beta$ and $\theta$, the average values of MSEs and the average length of 95%ACIs increase as $\tau$ grows.
iv. For fixed values of $\alpha , \beta$ and $\theta$, the average values of MSEs and the average length of 95%ACIs increase as CL increases.
v. The average value of estimates for $\beta$ and $\theta$ increases as the value of CL increases for fixed values of $\alpha , \beta$ and $\theta$, whereas the average value of estimates for $\alpha$ decrease.
vi. For fixed values of $\alpha , \beta$ and $\theta$, the mean result of estimates for $\beta$ and $\theta$ increase as the value of t rises, but the mean values of estimates for $\alpha$ decrease.

## Conclusions and suggestions for further studies

In this article, we used TRV modeling for SSPALT to estimate the unknown model parameters of the NH distribution using the MLE technique. It has been discovered that the MLEs for all unknown parameters cannot be derived explicitly. As a result, we utilized R software to compute MLEs numerically using the Optim() function. Using the observed Fisher information matrix, ACIs for the unknown parameters were also computed. An actual data set based on the timings of subsequent failures of sequential air conditioning system failures for each member of a Boeing 720 jet aircraft fleet was analyzed to illustrate the applicability of the different techniques. Finally, extensive simulation tests with various censoring mechanisms were carried out to evaluate the performance of the estimate procedure. In particular, MSEs, ACIs, and corresponding average interval lengths were used as benchmarks. According to our numerical findings, the values of MSEs and average lengths drop as sample size increases. Furthermore, when the censoring level is reduced, the considered estimates of $\alpha ,\beta$ and $\theta$ approach to their real values. As a future research, researchers may use rank set sampling to examine the NH distribution for hybrid censored data under SSPALT. A Bayesian analysis may be performed and compared with present study for the multiple censoring technique. Same has been added in the “Conclusion” section.

## Data Availability

All data available in the paper with references.
